# Interlinked Sister Chromosomes Arise in the Absence of Condensin during Fast Replication in *B. subtilis*

**DOI:** 10.1016/j.cub.2013.12.049

**Published:** 2014-02-03

**Authors:** Stephan Gruber, Jan-Willem Veening, Juri Bach, Martin Blettinger, Marc Bramkamp, Jeff Errington

**Affiliations:** 1 Max Planck Institute of Biochemistry, Research Group “Chromosome Organization and Dynamics,” Am Klopferspitz 18, 82152 Martinsried, Germany; 2 Molecular Genetics Group, Groningen Biomolecular Sciences and Biotechnology Institute, Centre for Synthetic Biology, University of Groningen, Nijenborgh 7, 9747 AG Groningen, The Netherlands; 3 Department of Biology I, Ludwig Maximilians University, Munich, Großhaderner Str. 2-4, 82152 Martinsried, Germany; 4 Centre for Bacterial Cell Biology, Institute for Cell and Molecular Biosciences, Newcastle University, Newcastle Upon Tyne NE2 4AX, UK

## Abstract

Condensin—an SMC-kleisin complex—is essential for efficient segregation of sister chromatids in eukaryotes [1, 2, 3, 4]. In *Escherichia coli* and *Bacillus subtilis*, deletion of condensin subunits results in severe growth phenotypes and the accumulation of cells lacking nucleoids [5, 6]. In many other bacteria and under slow growth conditions, however, the reported phenotypes are much milder or virtually absent [7, 8, 9, 10]. This raises the question of what role prokaryotic condensin might play during chromosome segregation under various growth conditions. In *B*. *subtilis* and *Streptococcus pneumoniae*, condensin complexes are enriched on the circular chromosome near the single origin of replication by ParB proteins bound to *parS* sequences [11, 12]. Using conditional alleles of condensin in *B. subtilis*, we demonstrate that depletion of its activity results in an immediate and severe defect in the partitioning of replication origins. Multiple copies of the chromosome remain unsegregated at or near the origin of replication. Surprisingly, the growth and chromosome segregation defects in rich medium are suppressed by a reduction of replication fork velocity but not by partial inhibition of translation or transcription. Prokaryotic condensin likely prevents the formation of sister DNA interconnections at the replication fork or promotes their resolution behind the fork.

## Highlights

• Smc-ScpAB inactivation causes a severe chromosome segregation defect in *B. subtilis*
• Replication origins remain interconnected in the absence of prokaryotic condensin
• Defects in chromosome segregation are highly dependent on growth conditions
• Reduction of replication fork velocity rescues segregation of replication origins

## Teaser

Gruber et al. show that conditional inactivation of prokaryotic condensin in *B. subtilis* results in immediate and severe defects in chromosome segregation under conditions promoting fast growth. The separation of replication origins is blocked in the absence of Smc-ScpAB but can be rescued by artificial reduction of replication fork speed.

## Results

### A Medium-Dependent Growth Defect in Condensin Mutants

**Figure 1:**
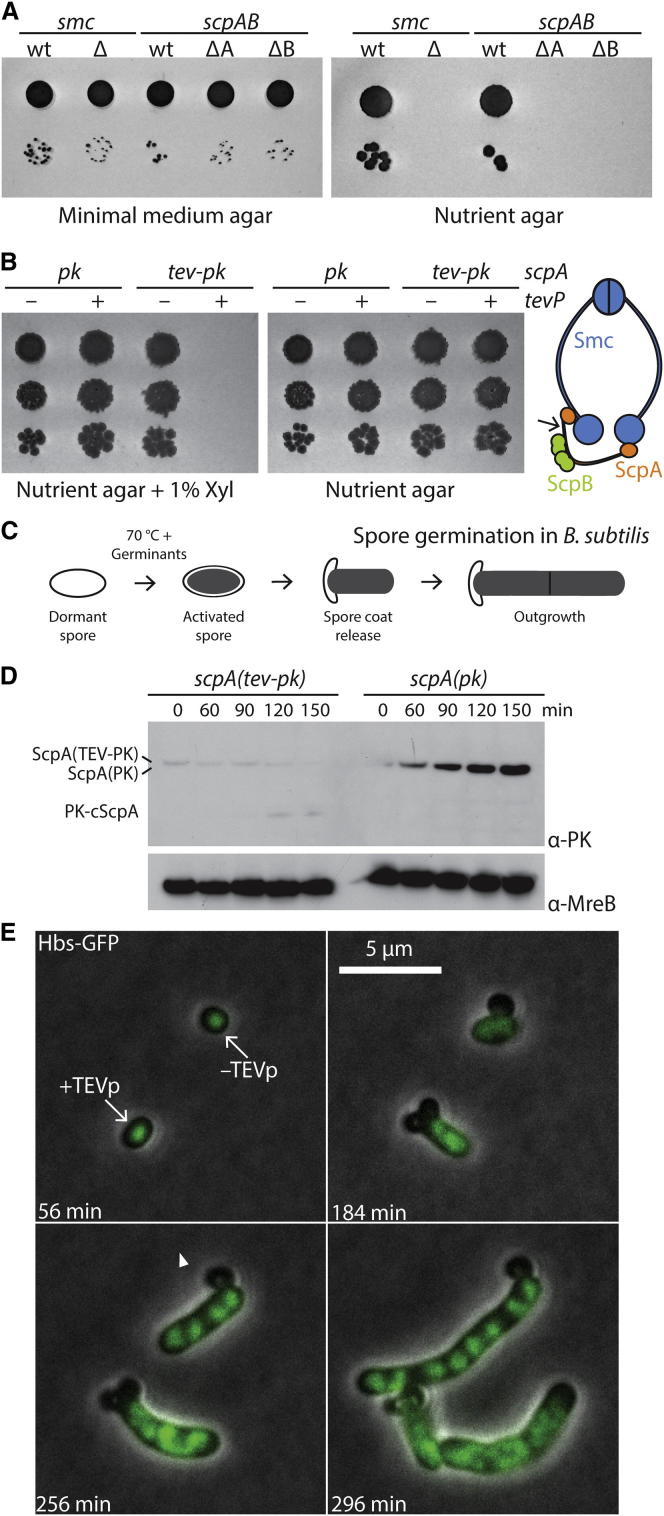
Conditional Inactivation of Condensin Blocks Nucleoid Segregation during Spore Germination (A) Colony formation of BSG1002 (*smc*^+^), BSG1007 (*Δsmc*), BSG1005 (*scpAB*^+^), BSG1004 (*ΔscpA*), and BSG1489 (*ΔscpB*) on SMG and NA plates at 37°C. Overnight cultures in SMG were diluted 81-fold and 60,000-fold. We spotted 5 μl of each dilution onto an agar plate. (B) Colony formation of BSG165 (*scpA(pk)*), BSG224 (*scpA(pk), P*_*xyl*_*-tevP*), BSG195 (*scpA(tev-pk)*), and BSG225 (*scpA(tev-pk), P*_*xyl*_*-tevP*) on nutrient agar at 37°C in the presence and absence of 1% xylose (left and right panels, respectively). Relevant genotypes of strains are indicated above the images: – and + denote the absence and presence, respectively, of the TEV protease gene (*tevP*); *pk* and *tev-pk* mark respective insertions in *scpA*. Serial dilutions of an overnight culture in LB were used as in (A). (C) Scheme of spore germination in *B. subtilis*. (D) TEV cleavage prevents accumulation of ScpA during spore outgrowth. Spores of strains BSG224 and BSG225 were heat activated for 30 min at 70°C and grown at 37°C. Samples were taken every 30 min for the preparation of whole-cell extracts. Proteins were immunoblotted using antibodies directed against the PK epitope tag and MreB protein. (E) Time-lapse microscopy of an equal mixture of germinating spores of strains BSG221 (*scpA(tev-pk), hbs-gfp*) and BSG222 (*scpA(tev-pk), P*_*xyl*_*-tevP, hbs-gfp*). Spores displaying wild-type and mutant phenotypes are labeled as –TEVp and +TEVp, respectively. Cells were incubated on LB agar pads supplemented with glucose and xylose to induce TEVp expression (LBG) at 30°C. Hbs-GFP and phase-contrast signals are shown in green and gray colors, respectively. Outgrowing spores harboring *tevP* and *hbs*-*gfp* display an unusually large diameter. This seems to be caused at least in part by Hbs-GFP, because unlabeled cells or cells labeled otherwise do not show this pronounced shape defect.

Replication forks proceed with remarkable velocities in bacteria—up to ∼1,000 bases/s. Even so, during fast growth the time taken to replicate a chromosome, called the C period, often exceeds the generation time and thus becomes potentially limiting for cell growth. To bypass this potential limitation, bacteria reinitiate replication at the origin of replication (*oriC*) prior to completion of previous rounds so that several forks are operating on each half of the chromosome at any one time [13, 14, 15]. Prokaryotic condensin forms asymmetric rings comprised of Smc, ScpA, and ScpB subunits [16] that have been implicated in promoting the segregation of chromosomes, especially during multifork DNA replication [17]. To revisit the condensin phenotype in *Bacillus subtilis*, we generated null mutants of *smc*, *scpA*, and *scpB* genes by making in-frame deletions and thus avoiding polar effects on neighboring genes. These mutants could readily be isolated at 37°C on minimal medium supplemented with glucose and glutamate (SMG). They formed colonies with normal efficiency at 37°C and also at decreased or elevated temperatures, but failed to do so on rich medium even at low temperatures (Figure 1A; see also Figure S1A available online). These results indicate that the growth defect of condensin mutants is strongly media dependent but not strictly correlated with either temperature or growth rate, thus questioning the notion that high numbers of origins per chromosome might be the cause of *Δsmc* lethality.

### Depletion of Condensin Blocks Chromosome Segregation in Rich Medium

To study the direct consequences of loss of prokaryotic condensin and to avoid any effects due to suppressor mutations, we built a conditional construct, ScpA(TEV-PK), in which TEV cleavage sites and a PK epitope tag were inserted into the central region of ScpA. Expression of TEV protease (TEVp) from the xylose promoter in ScpA(TEV-PK) cells prevented formation of colonies, implying that ScpA is efficiently inactivated by TEV protease (Figure 1B). To eliminate condensin prior to any ongoing rounds of DNA replication, we used germinating spores of *B. subtilis* (Figure 1C) [18, 19]. Accumulation of ScpA(TEV-PK) during spore germination was efficiently prevented by expression of TEVp (Figure 1D). We initially investigated nucleoid architecture and distribution in these cells using an ectopically expressed Hbs-GFP fusion protein as a general label for DNA [20]. A mixture of *scpA*(*tev*-*pk*) spores encoding or lacking TEVp were heat activated to induce spore germination and immobilized on agar pads. Outgrowth of spores was then imaged by time-lapse fluorescence microscopy. Cells lacking TEVp released their spore coat ∼90–120 min after heat activation and formed chains of rod-shaped cells comprising uniformly sized and distributed nucleoids (Figure 1E, top cell). TEVp-containing spores displayed similar outgrowth kinetics and initially exhibited comparable Hbs-GFP signal. However, even at the early stages of outgrowth, cells failed to form individual nucleoids and generally displayed an apparently unseparated mass of DNA that was unevenly distributed throughout most of the cell.

**Figure 2:**
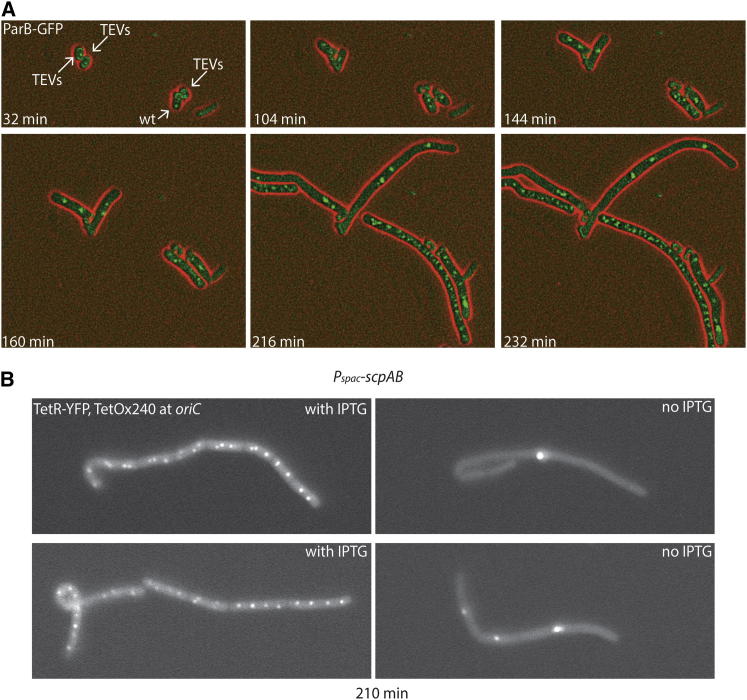
Inactivation of Smc-ScpAB Prevents Segregation of *oriC* (A) Time-lapse microscopy of an equal mixture of germinating spores of strains BSG203 (*P*_*xyl*_*-tevP, parB-gfp*) and BSG204 (*scpA(tev-pk), P*_*xyl*_*-tevP, parB-gfp*). Spores encoding wild-type ScpA and ScpA containing a TEV site are labeled as WT and TEVs, respectively. LBG agar pads were incubated at 37°C. ParB-GFP and phase-contrast signals are shown in green and red colors, respectively. (B) Germinating spores of strain BSG239 (*P*_*spac*_*-scpA(tev-pk)scpB, P*_*xyl*_*-tevP, parB::tetOx240, tetR-yfp*) grown in liquid LBG media in the presence and absence of 1 mM isopropyl-thiogalactopyranosid (IPTG) at 37°C. YFP images were taken 210 min after heat activation of spores.

To visualize the early stages of chromosome segregation, we next specifically labeled the *oriC* region by fusing *B. subtilis* ParB protein to GFP. We mixed spores with or without TEV sites in ScpA and followed their outgrowth with time-lapse microscopy. Shortly after release of the spore coat, ScpA(PK) cells displayed a second focus of ParB-GFP (104 min), and this quickly became a multiplicity of foci that were uniformly distributed along the length of the cell (Figure 2A, spore labeled as wild-type [WT]). In the majority of ScpA(TEV-PK) cells (labeled as TEVs), in contrast, the focus of ParB-GFP showed little sign of splitting. The ParB-GFP foci generally stayed low in number but instead appeared increasingly bright, suggesting that multiple *oriC* regions are present but remain in close proximity. Indeed, we found that replication initiation was largely unaffected by ScpA cleavage at early stages of spore outgrowth, as judged with marker frequency analysis (Figure S2D). Repression of *scpAB* expression using a *P*_*spac*_ promoter and of *smc* using a *P*_*xyl*_ promoter in both cases had a severe effect on *oriC* segregation during spore outgrowth, similar to the TEV cleavage of ScpA (Figures S2A and S2B). Chromosomes labeled near *oriC* by TetR-YFP (FROS) instead of ParB-GFP also exhibited failed segregation in cells lacking normal levels of ScpA and ScpB (Figure 2B).

Next, we monitored the region around the terminus of replication (*ter*) using the replication terminus protein (RTP) fused to GFP in cells synthesizing ParB-mCherry. Interestingly, the number and distribution of RTP foci were only mildly compromised by repression of *scpAB* expression at early stages of outgrowth (Figure S2C). This indicates that the defect in separation of sister chromosomes might be most pronounced close to the origin of replication, where Smc-ScpAB is highly enriched in cells of *B. subtilis* [11, 12].

### Slowing of Replication Forks but Not Growth Rate Per Se Suppresses Lethality of *Δsmc* Mutants

**Figure 3:**
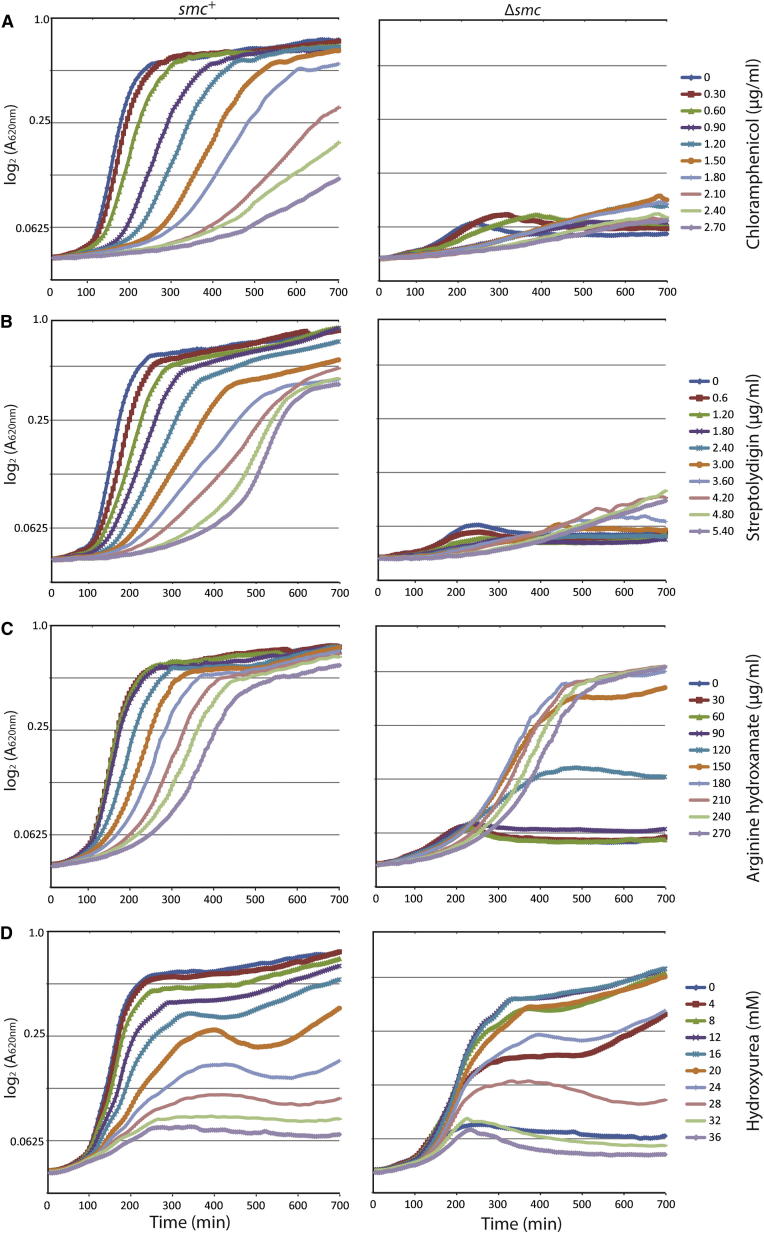
Rescue of Lethal Growth Defect of *Δsmc* by Small Molecules Growth of BSG1002 and BSG1007 in microtiter plates containing LB medium supplemented with different concentrations of the following antibiotics: (A) Chloramphenicol. (B) Streptolydigin. (C) Arginine hydroxamate. (D) Hydroxyurea. Growth at 37°C was monitored by light scattering at a wavelength of 620 nm.

We next wondered why proliferation and chromosome segregation are blocked in rich medium in condensin mutants but appear largely unperturbed in minimal medium. Supposing that condensin organizes and separates multiple origins present in nucleoids under rich medium conditions, then artificially reducing the growth rate and thus the rate of replication initiation might suppress the condensin phenotype in rich medium. To test this, we screened for small-molecule inhibitors that decrease the growth rate of *B. subtilis* in Luria Bertani (LB) medium in a dosage-dependent manner. Addition of chloramphenicol or streptolydigin, inhibitors of translation and transcription, respectively, produced exponentially growing cultures of wild-type cells with doubling times ranging from 18 to 80 min (Figures 3A and 3B). Cultures of *Δsmc* cells, however, were unable to grow in LB medium supplemented with either chloramphenicol or streptolydigin at any tested concentration. Thus, rapid growth and multifork replication appear not to be directly related to the growth phenotype of *Δsmc* mutants.

We then speculated that the abundance of amino acids in LB medium might play a role in causing the defect in chromosome segregation. To test this, we reduced the availability of a single amino acid using arginine hydroxamate (Rhx). Rhx leads to activation of the stringent response by specifically inhibiting the synthesis of arginyl-tRNA. Interestingly, *Δsmc* cells showed robust growth at 150 μg/ml Rhx or more (Figure 3C). This finding implied that partial activation of the stringent response is sufficient to rescue the growth of cells lacking condensin in rich medium. In *B. subtilis*, activation of the stringent response has a direct impact on replication fork progression through its inhibition of DNA primase, DnaG, mediated by the alarmone ppGpp [21]. Therefore, the need for Smc-ScpAB in segregation of sister DNA molecules might depend on the velocity of replication fork progression. Hydroxyurea (HU) slows down incorporation of nucleotides into DNA by decreasing the cellular pool of dNTP via inhibition of ribonucleotide reductase [22]. Intriguingly, addition of HU to LB medium significantly improved the growth of *Δsmc* strains at concentrations as low as 4 mM (Figure 3D). The same concentrations had very little effect on the growth of wild-type cells, indicating that *smc* mutants can segregate their chromosomes under rapid growth conditions, provided that DNA replication has been artificially slowed. HB-EMAU, an inhibitor of DNA polymerase PolC in *B. subtilis*, also partially rescued growth of *Δsmc* cells (Figure S3B) [23]. Importantly, the activation of the DNA damage response is neither necessary nor sufficient for suppression of *Δsmc* lethality by HU (Figure S3C) [24, 25]. Together, these findings support the view that lowering replication fork velocity, but not slowing cell growth, bypasses the strict requirement for Smc in chromosome segregation in rich medium.

### Chromosome Segregation Occurs Almost Normally in *Δsmc* When Replication Fork Velocity Is Reduced

**Figure 4:**
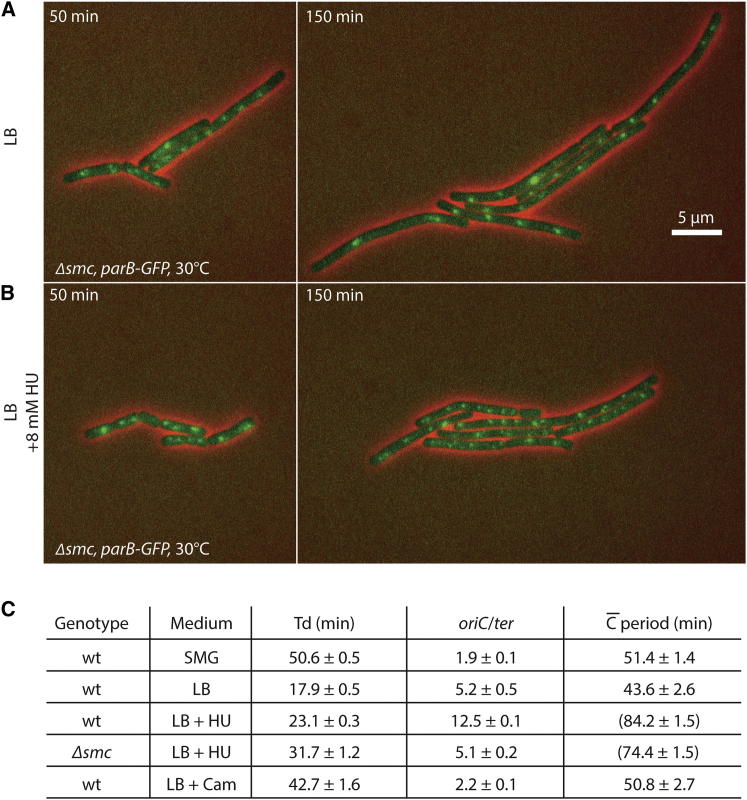
Rescue of the Segregation of *oriC* at Artificially Low Replication Fork Velocities (A and B) Time-lapse microscopy of strains BSG448 (*parB-gfp, Δsmc*) grown on LB agar pads at 30°C in the absence (A) and presence (B) of 8 mM hydroxyurea. ParB-GFP and phase-contrast signals are shown in green and red colors, respectively. Corresponding images of cells with a wild-type *smc* allele are shown in Figure S4. (C) Growth and replication characteristics of BSG1002 and BSG1007 at 37°C. Cells were grown to exponential phase in SMG and diluted 1:11 into SMG medium or LB medium with 8 mM HU, with 1.2 μg/ml chloramphenicol, or without inhibitors. Doubling times (Td) and *oriC*/*ter* ratios were determined experimentally. Standard deviation from mean values was calculated from at least three independent replicates. Population average replication periods were estimated on the assumption of uniform replication of the chromosome and balanced growth based on the following equation [26]: C_(min)_ = ln(*oriC*/*ter*)/ln(2) × Td_(min)_. Estimates derived for cells displaying potentially nonuniform DNA replication and/or unbalanced growth under the given conditions are shown in parenthesis.

To directly test whether addition of HU permits more efficient *oriC* segregation in *Δsmc* cells, we visualized ParB-GFP dynamics in LB medium by using time-lapse microscopy. As expected, wild-type cells grew well and segregated ParB-GFP foci efficiently either in the presence or absence of HU (Figures S4A and S4B). *smc* mutant cells, however, contained slightly fewer foci after 50 min on LB agar pads, and, consistent with our previous findings with germinating spores, the number of foci remained almost unchanged 100 min later. Importantly, in the presence of 8 mM HU, the number of ParB-GFP foci increased significantly during incubation (Figures 4A and 4B). Thus, mild inhibition of the synthesis of deoxynucleotides enables cells lacking Smc-ScpAB to segregate their *oriC* regions with apparently normal efficiency.

Finally, we estimated the average time taken to replicate a chromosome under various conditions by determining growth rates and *oriC*/*ter* ratios (Figure 4C) [26]. Wild-type cells exhibited an almost 20% longer replication period in SMG compared to LB medium. The addition of HU resulted in significant overinitiation of DNA replication, as judged by quantitative PCR and whole-genome sequencing (Figures 4C and S4C) [22]. Assuming uniform replication of the chromosome in the presence of HU, this translates into an ∼2-fold increase in replication period in wild-type cells. *smc* mutant cells grown in HU displayed a replication period similar to wild-type but a significantly lower *oriC*/*ter* ratio, indicating a delay in replication initiation possibly caused by the origin segregation failure. A similar trend was observed at later stages of spore germination upon depletion of *scpAB* (Figure S2D). A lower number of origins per chromosome in turn might result in higher replication fork speed due to slower consumption of nucleotides by fewer replication forks (Figure 4C). Thus, any initial failure in *oriC* segregation could increase the burden on later rounds of replication and segregation, providing a possible explanation for the drastic phenotypic differences in *Δsmc* cells grown in SMG and LB medium despite the relatively small difference in the replication periods.

## Discussion

### Smc-ScpAB Promotes Resolution of Sister Chromosomes

Bacterial SMC complexes have been proposed to delay or promote immediate DNA segregation behind the replication fork by analogy to eukaryotic cohesin or condensin, respectively. Our data demonstrate that the partitioning of nucleoids—especially near *oriC*—is severely blocked in the absence of any of the three subunits of Smc-ScpAB. Promoting resolution of sister DNA molecules might thus be the primordial function of SMC-kleisin complexes, which has been evolutionarily maintained in prokaryotic condensin, as well as condensin in eukaryotes. Interestingly, global levels of chromosome condensation are apparently not affected by the absence of Smc (Figure 1E). If prokaryotic condensin indeed compacts chromosomal DNA, it probably does so locally: for example, in a region around the origin of replication, where it is most enriched via ParB/*parS*.

### Linkages between Replication Origins

Our findings suggest that persistent linkages between sister chromosomes are created at replication origins in the absence of prokaryotic condensin, especially during fast DNA replication. What might be the nature of these linkages? Both DNA intertwining and bridges mediated by proteins are known to hold sister chromatids together in eukaryotes [27, 28]. In *E. coli*, reduced or increased levels of topoisomerase IV activity hinder or promote sister segregation, respectively [29], suggesting that removal of DNA intertwining is critical for the timing of chromosome segregation. The SeqA protein has recently been implicated in mediating temporary sister chromosome cohesion behind the replication fork in *E. coli* [30]. As-yet-unidentified proteins might similarly hold chromosomes together in *B. subtilis*, possibly in combination with DNA intertwining.

### A Link between Replication Speed and Sister DNA Connection

Our results suggest that quickly replicating chromosomes pose significant challenges during segregation, making *oriC* partitioning absolutely dependent on condensin. Fast DNA replication might increase the burden on DNA topoisomerase, thus leading to more sister DNA intertwining. Condensin has indeed previously been linked to sister DNA decatenation in prokaryotes and eukaryotes [4, 31]. In *E. coli*, condensin interacts directly with the decatenating enzyme, DNA topoisomerase IV [32, 33]. Surprisingly, however, David Rudner and colleagues found that inactivation of topoisomerase IV does not severely impair segregation of replication origins in *B*. *subtilis* [34], probably indicating that DNA intertwining does not hinder segregation of replication origins in *B*. *subtilis*. Fast DNA replication might instead (or in addition) generate long stretches of juxtaposed sister DNA, which serve as platforms for nucleoid-associated proteins to stably bridge sister chromosomes. Condensin might rapidly organize freshly replicated sister DNA into separate entities—via lengthwise condensation—thereby minimizing the potential for sister DNA contacts.

### Conclusions

The precise physiological role of SMC-kleisin complexes in bacteria is poorly understood at the moment. Here, we find that condensin is absolutely essential for the separation of sister DNA close to the origin of replication, when cells are grown in rich medium. Strikingly, the defects in chromosome segregation and proliferation are alleviated when the velocity of replication forks is reduced using small-molecule inhibitors. We conclude that chromosome partitioning in *B. subtilis* is strictly dependent on Smc-ScpAB when chromosomes are replicated by quickly progressing forks. SMC-kleisin complexes might have emerged to resolve proteinaceous or DNA-based interconnections between sister DNA molecules, thereby allowing the evolution of faster DNA replication.

## Supplemental Information

Document S1. Supplemental Experimental Procedures and Figures S1–S4
