# Supplementary material for: Interlinked Sister Chromosomes Arise in the Absence of Condensin during Fast Replication in B. subtilis
Source: Curr Biol. 2014 Feb 3;24(3):293–8. doi: 10.1016/j.cub.2013.12.049 (PMC3919155; doi:10.1016/j.cub.2013.12.049)
Supplement: Document S1. Supplemental Experimental Procedures and Figures S1–S4 [file mmc1.pdf]

Current Biology, Volume *24*

Supplemental Information

Interlinked Sister Chromosomes Arise in the Absence of Condensin during Fast Replication in *B. subtilis*

Stephan Gruber, Jan-Willem Veening, Juri Bach, Martin Blettinger, Marc Bramkamp, and Jeff Errington

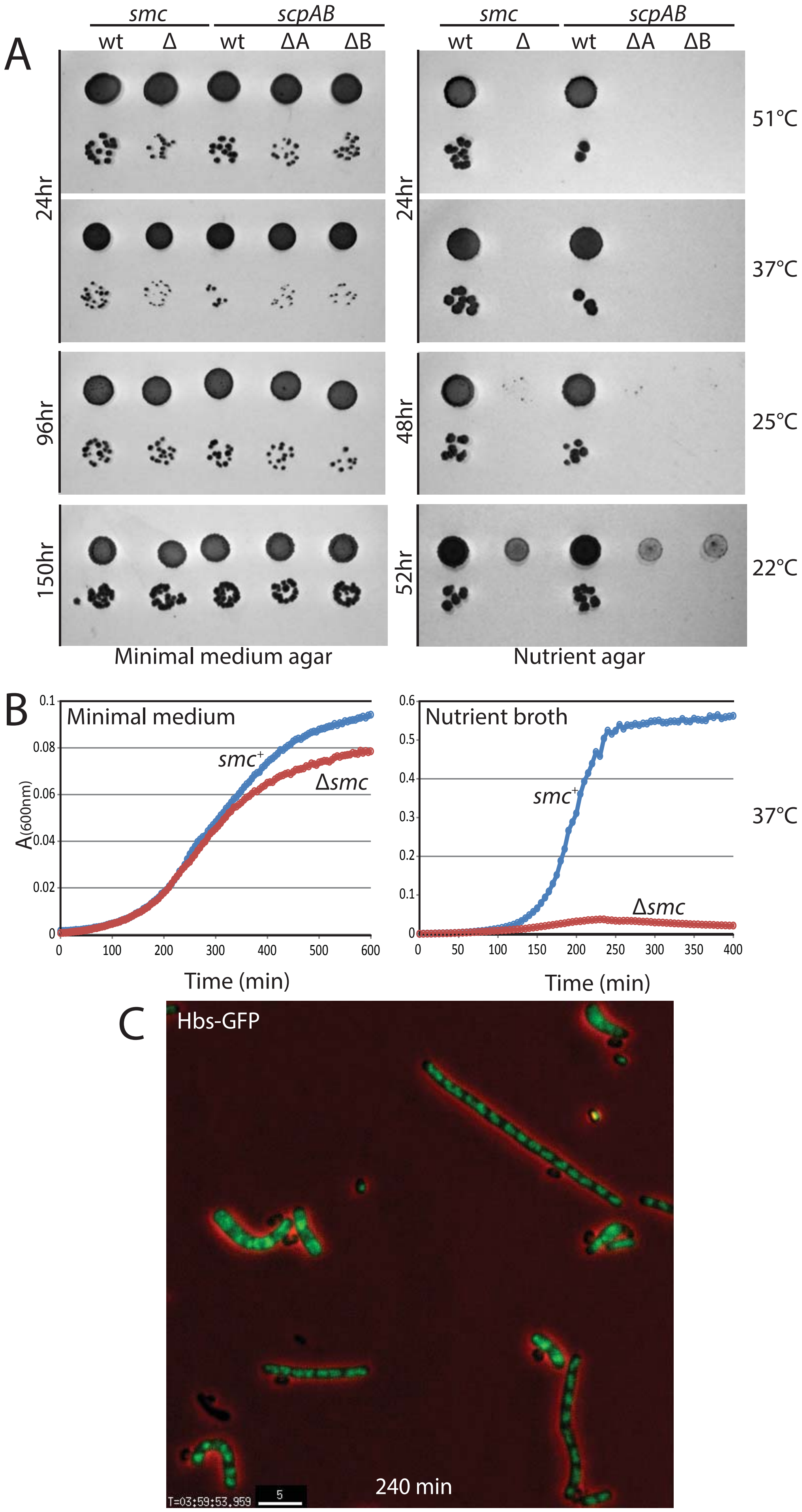
A
smc
scpAB
wt
Δ
wt
ΔA
ΔB
24hr
96hr
150hr
48hr
52hr
51°C
37°C
25°C
22°C
Minimal medium agar
Nutrient agar
B
Minimal medium
Nutrient broth
A(600nm)
smc+
Δsmc
37°C
Time (min)
C
Hbs-GFP
240 min
T=03:59:53.959
5

Figure S1

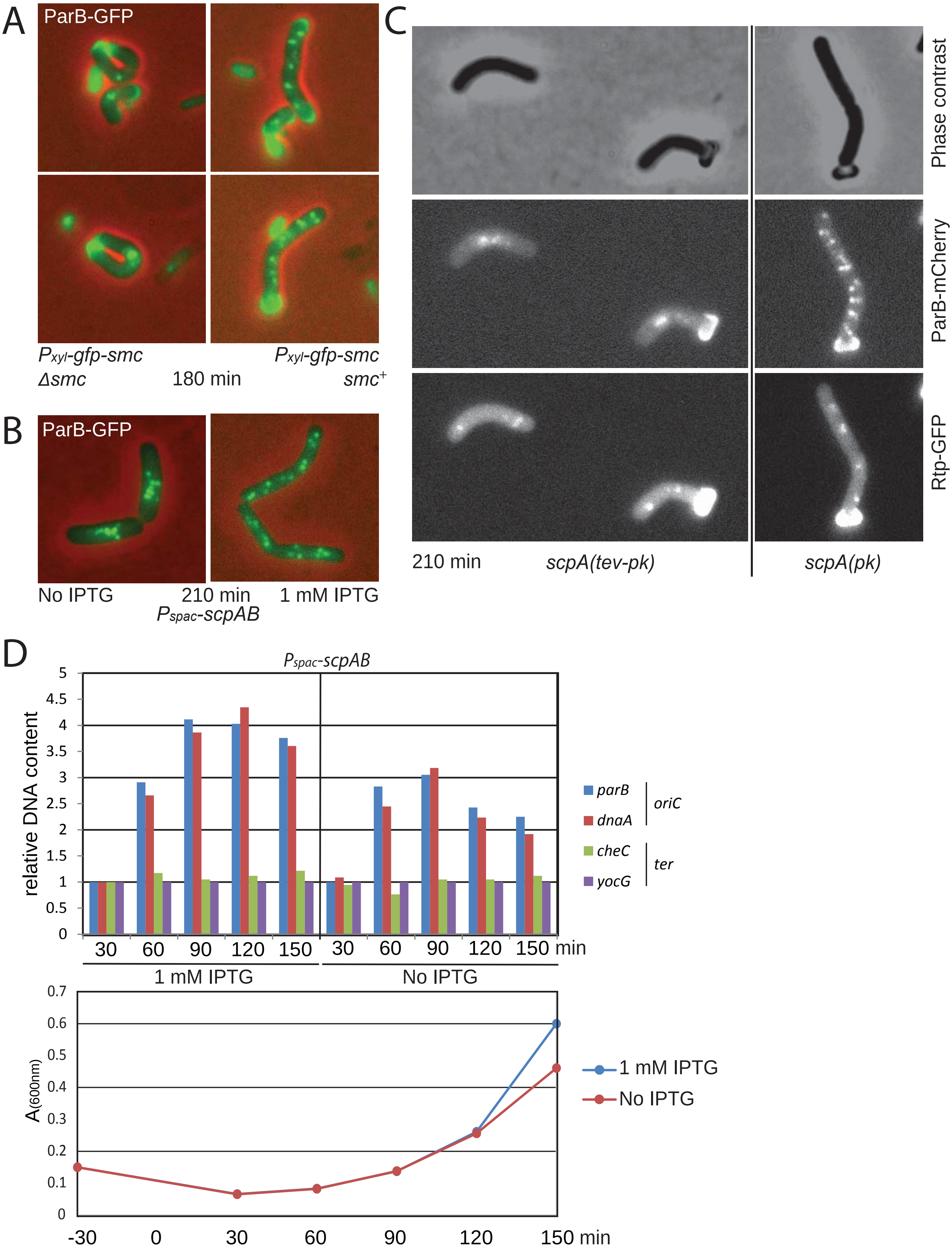
A
ParB-GFP
Pxyl-gfp-smc
Δsmc
180 min
Pxyl-gfp-smc
smc+
B
ParB-GFP
No IPTG
210 min
1 mM IPTG
Pspac-scpAB
C
Phase contrast
ParB-mCherry
Rtp-GFP
210 min
scpA(tev-pk)
scpA(pk)
D
Pspac-scpAB
relative DNA content
5
4.5
4
3.5
3
2.5
2
1.5
1
0.5
0
30
60
90
120
150
30
60
90
120
150 min
1 mM IPTG
No IPTG
parB
dnaA
oriC
cheC
yocG
ter
A(600nm)
0.7
0.6
0.5
0.4
0.3
0.2
0.1
0
-30
0
30
60
90
120
150 min
1 mM IPTG
No IPTG

Figure S2

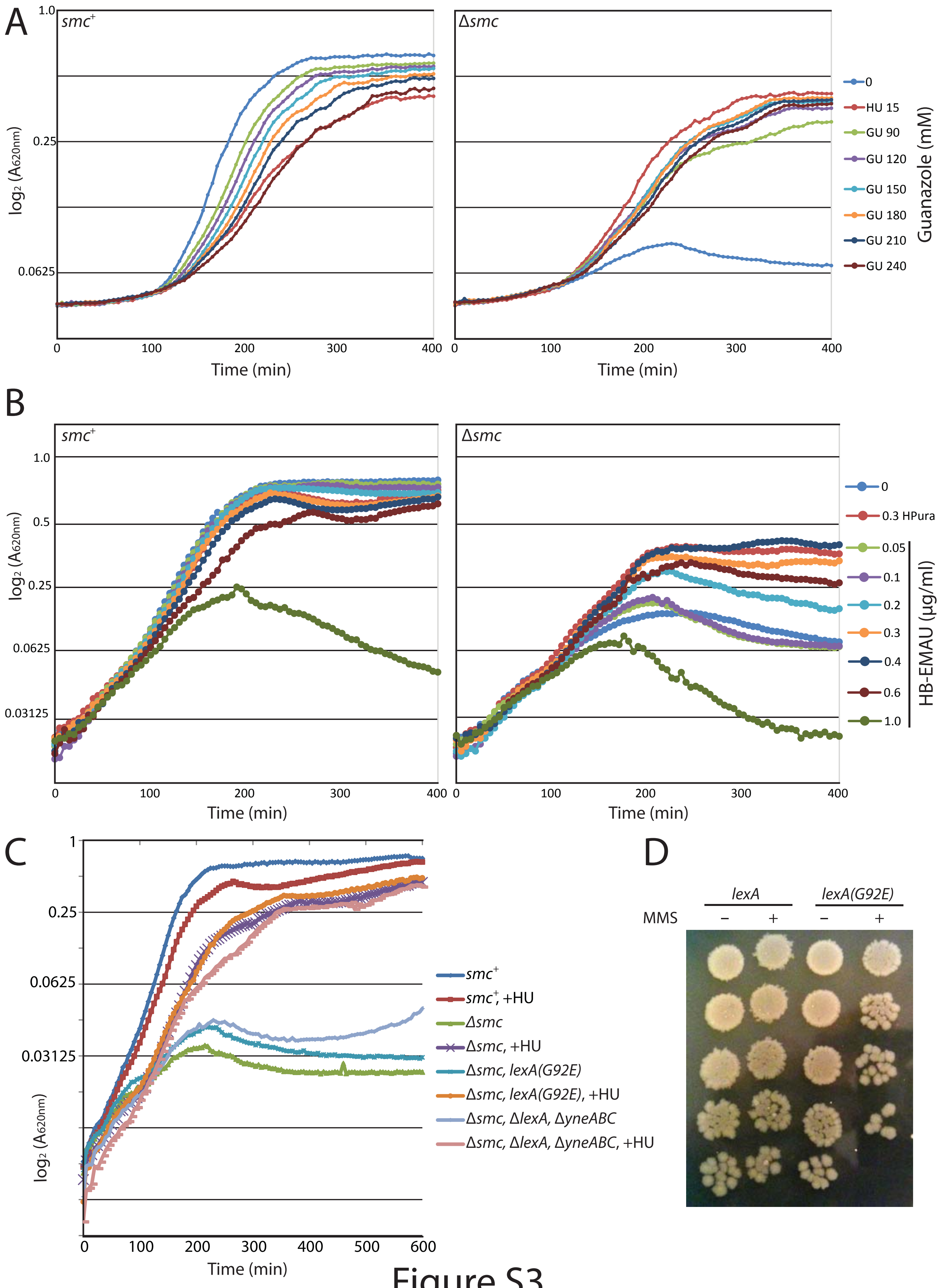

A
smc+
Δsmc
log2 (A620nm)
1.0
0.25
0.0625
Time (min)
0
HU 15
GU 90
GU 120
GU 150
GU 180
GU 210
GU 240
Guanazole (mM)
B
0.5
0.03125
0.3 HPura
0.05
0.1
0.2
0.3
0.4
0.6
1.0
HB-EMAU (µg/ml)
C
smc+, +HU
Δsmc
Δsmc, +HU
Δsmc, lexA(G92E)
Δsmc, lexA(G92E), +HU
Δsmc, ΔlexA, ΔyneABC
Δsmc, ΔlexA, ΔyneABC, +HU
D
lexA
lexA(G92E)
MMS

Figure S3

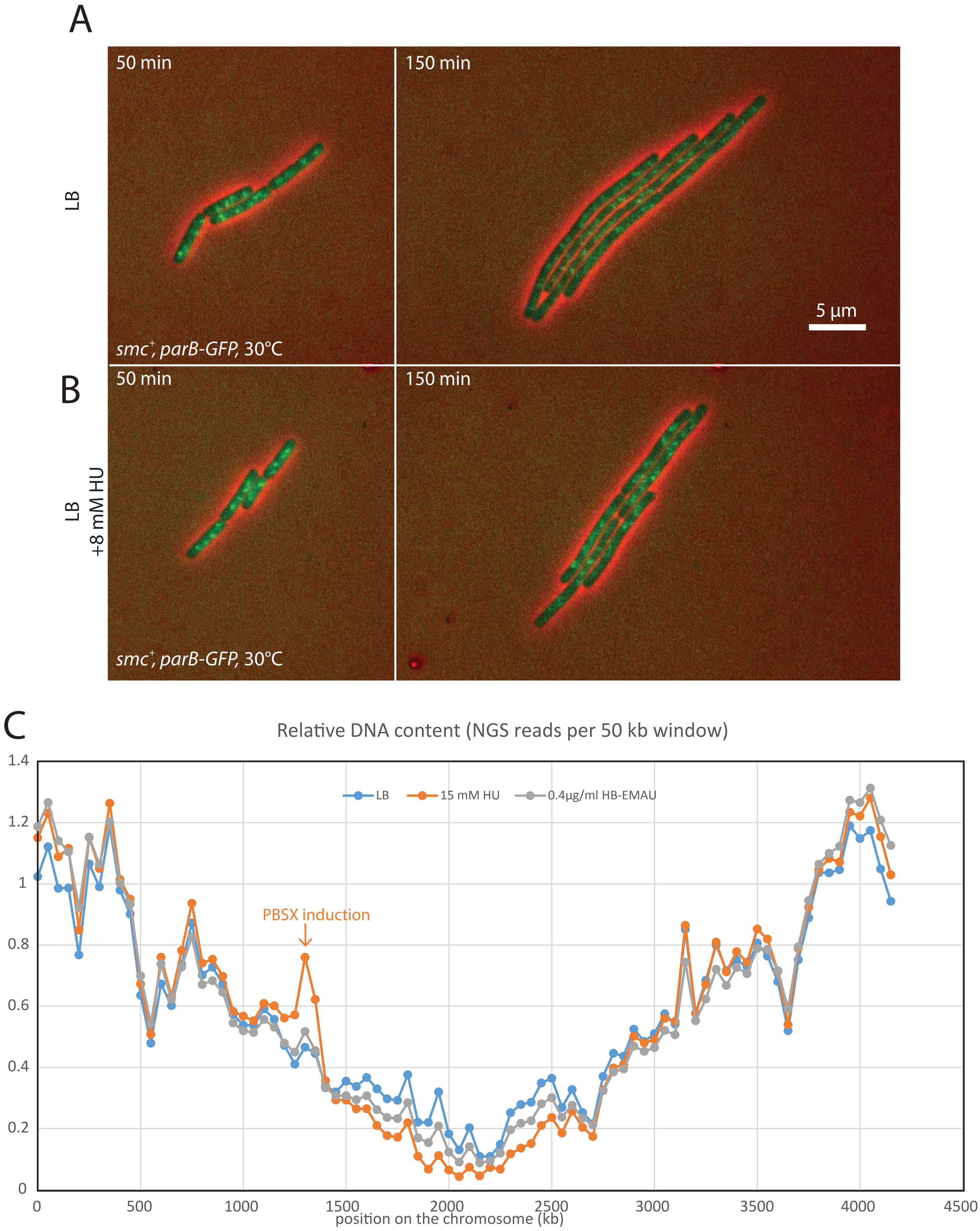
A
50 min
150 min
LB
5 µm
smc+, parB-GFP, 30°C
B
50 min
150 min
LB
+8 mM HU
smc+, parB-GFP, 30°C
C
Relative DNA content (NGS reads per 50 kb window)
LB
15 mM HU
0.4µg/ml HB-EMAU
PBSX induction
position on the chromosome (kb)
1.4
1.2
1
0.8
0.6
0.4
0.2
0
0
500
1000
1500
2000
2500
3000
3500
4000
4500

Figure S4

Supplemental Figure Legends

Fig. S1 Relates to Figure 1. Condensin depletion impairs growth and prevents nucleoid division.

(A) Colony formation of BSG1002 (*smc*$^+$), BSG1007 (*Δsmc*), BSG1005 (*scpAB*$^+$), BSG1004 (*ΔscpA*) and BSG1489 (*ΔscpB*) on SMG and NA plates at four different temperatures. Overnight cultures in SMG were diluted 81 and 60,000 fold. 5 µl of each dilution was spotted onto an agar plate. The 37 °C panel is identical to the one shown in Fig. 1A. After prolonged incubation at 22 or 25 °C low numbers of colonies of *smc*, *scpA* or *scpB* mutants appear on nutrient agar. Considering their relatively low plating efficiency and slow growth those colonies likely originate from cells harboring suppressor mutations.

(B) Growth of BSG1002 and BSG1007 in liquid SMG medium and nutrient broth. Cultures growing exponentially in SMG medium were diluted eleven-fold into fresh medium at time point zero and grown in microtitre plates with continuous shaking.

(C) Same experiment as displayed in Fig. 1E showing a larger field of cells after 240 min of incubation.

Fig. S2 Relates to Figure 2. Depletion of condensin subunits prevents segregation of *oriC*.

(A) Germinating spores of strain BSG299 (P_{xyl}*-gfp-smc, Δsmc, parB-gfp*) and BSG300 (P_{xyl}*-gfp-smc, smc*$^+$*, parB-gfp*) grown in liquid LBG media in the absence of xylose at 37 °C. GFP and phase contrast images were taken 180 minutes after heat activation of spores. Note that the released spore coat frequently stays associated with cell poles and exhibits high auto-fluorescence under these conditions.

(B) Spores of strain BSG363 (*parB-gfp*, P_{spac}-*scpAB, Δ7-parS*) were germinated in LBG medium at 37 °C with or without 1 mM IPTG. GFP and phase contrast images were taken 210 min after heat activation.

(C) Spores of BSG222 (*scpA(pk),* P_{xyl}*-tevP, rtp-gfp, parB-mCherry*) and BSG223 (*scpA(tev-pk),* P_{xyl}*-tevP, rtp-gfp, parB-mCherry*) were heat activated and germinated in LBG medium in the presence of xylose at 30 °C. GFP, mCherry and PC images were taken 210 min after heat activation. Note that spore coats frequently stay attached to cell poles after germination and exhibit high auto-fluorescence in the GFP and mCherry channels.

(D) Marker frequency analysis during spore germination. Spores of strain BSG282 (parB-GFP, P_{spac}-*scpAB*) were germinated in the presence or absence of 1 mM IPTG. Samples were taken every 30 min for OD_{600} measurements (bottom panel) and gDNA preparation. gDNA samples were diluted to 1 ng/µl and analyzed by qPCR using two primer pairs for the *oriC* and for the *ter* regions. Amount of DNA is indicated relative to the *yocG* DNA (top panel).

Fig. S3 Relates to Figure 3. Artificial slowing of replication forks rescues growth of *smc* mutants in LB medium independent of SOS.

(A, B) Growth analysis of BSG1002 and BSG1007 in microtitre plates at 37 °C as in Fig. 3 using different concentrations of guanazole (A) and HB-EMAU (B).

(C) Growth of BSG2 (*smc*$^{+}$), BSG68 (*Δsmc*), BSG437 (*lexA*(G92E), *Δsmc*) and BSG439 (*ΔlexA, ΔyneABC*, *Δsmc*) in LB medium supplemented with or without 15 mM HU. Cells were grown at 37 °C.

(D) SOS response in wt and *lexA*(G92E) cells. Colony formation of BSG430 (wt) and BSG432 (*lexA*(G92E)) after exposure to MMS (10 mM in SMG for 30 min at 37 °C) was tested by 5-fold serial dilution and spotting onto nutrient agar plates.

Fig. S4 Relates to Figure 4. Effect of HU on origin segregation and DNA replication in wild-type cells.

(A) Cells of BSG447 were grown in SMG medium in exponential phase and spotted onto LB agar pads and incubated at 30 °C in a temperature chamber at the microscope. Images were taken 50 and 150 min after spotting. ParB-GFP and phase contrast signals are shown in green and red colors, respectively.

(B) As in (A) with 8 mM HU.

(C) Whole genome sequencing of chromosomes isolated under different growth conditions. Cells of BSG1001 (wt) were grown in LB medium in exponential phase at 37 °C. 15 mM HU or 0.4 μg/ml HB-EMAU were added for 1 hr and genomic DNA was isolated and analyzed by Illumina shotgun sequencing. Individual reads were mapped to 50 kb windows of the *B. subtilis* genome and displayed in percentage of the total number of reads per sample per sliding window. Coordinates are based on the *B. subtilis* genome sequence (GenBankID AL009126.3; *oriC* is at 0 Mb). Exposure to HU and HB-EMAU leads to slight over-initiation of DNA replication and underrepresentation of an approximately 1Mb region around *ter* (around position 2.2 Mb). Note that exposure to HU leads to the induction of the replication of the prophage PBSX under these conditions in a small sub-population of cells (indicated by arrow). Assuming fully-induced PBSX would result in about 50 copies per cell [S1, S2], the observed less than twofold increase of prophage sequences would indicate activation of PBSX in about 4 % of cells.

Supplemental Experimental Procedures

Bacterial growth and strain construction

Bacillus subtilis strains used in this study are derivative of 168ED and 1A700. Relevant genotypes are given in figure legends. Spizizen minimal salt medium [S3] (supplemented by 5 g/l glucose, 1 g/l glutamate and 20 mg/l tryptophan), Luria Bertani medium ("LB-Miller") and nutrient broth (Oxoid, UK) were used for growth in liquid culture. 20 g/l BactoAgar (Difco) was added for growth in petri dishes. Expression from P_{spac} and P_{xyl} were induced by 1 mM IPTG and 10 g/l xylose, respectively. Gene deletion and tagging in *B. subtilis* was performed via double cross-over recombination using targeting constructs cloned in *E. coli*. Sequences of constructs are available upon request.

For deletion of *smc* the selection marker *ermB* was cloned downstream of the *smc-ftsY* operon [S3] and a large part of the *smc* coding sequence (19-2730 bp) was deleted. To create *ΔscpA* and *ΔscpB* mutants a spectinomycin resistance cassette was cloned upstream of the operon [S3] and regions of *scpA* (43-690 bp) and *scpB* (13-573 bp) were deleted, respectively. ScpA(PK) and ScpA(TEV-PK) were generated analogously by insertion of following amino acid sequences after ScpA(L88): TR-GIPNPLLGLD-GIPNPLLGLD-GIPNPLLGLD-GSSS and TS-ENLYFQG-PR-ENLYFQG-GS-ENLYFQG-TR-GIPNPLLGLD-GIPNPLLGLD-GIPNPLLGLD-GSSS. The *scpAB* operon was put under control of the P_{spac} promoter by insertion of a fragment from plasmid pAPNC213cat – comprising *cat, lacI*, and P_{spac} – upstream of *scpAB*. The *gfp-smc* fusion under control of the P_{xyl} promoter has been reported previously [S4]. *tevP*(S219V) was fused to the P_{xyl} promoter from plasmid pSG1154 and cloned into plasmid pDG3661 and pDG1664, which integrate by double cross-over into the *amyE* and *thrC* locus, respectively. Hbs-GFP was expressed from the native *hbs* promoter and integrated ectopically at the nonessential *amyE* locus. ParB-GFP and ParB-mCherry fusion proteins and the TetR-YFP labeling system were described previously [S5, S6]. The G92E mutations in *lexA* was cloned in *E. coli* and introduced into *B. subtilis* (*trpC2*) by congression using selection with wild-type *trpC* marker cloned into a plasmid in *E. coli*. *ΔlexA* and *ΔyneABC* deletions were kindly provided by Yoshikazu Kawai [S7].

Imaging of spore germination and outgrowth

For spore preparation cells were grown exponentially at 30°C in CH medium supplemented by 0.1 mM IPTG when needed. Cells were harvested by centrifugation, resuspended in sporulation medium (supplemented by tryptophan and threonine as needed) and incubated at 37°C for 24 hr. Spores were then pelleted, treated with lysozyme (1 mg/ml) for 1 hr at 37 °C to remove vegetative cells and washed extensively in water. Purified spores were kept at 4 °C for up to two weeks. Spore germination was induced by resuspension of spores in LB medium with 200 mg/ml alanine and 5 g/l glucose or SMG medium supplemented with 200 mg/l alanine and 200 mg/l casamino acids to an OD_{600} of 0.5 and heat treatment for 30 min at 70 °C. The spore suspension was diluted twofold in medium pre-warmed at 30 or 37 °C. For time-lapse imaging spores or spore mixtures were then spotted onto pre-warmed agarose pads (1.5 %) containing medium supplemented with 1 % xylose or 1 mM IPTG as needed. Images were taken every 8 min min on a Deltavision (Applied Precision) IX71Microscope (Olympus) using a CoolSNAP HQ camera (Princeton Instruments) with a 63x 1.40 NA phase contrast objective (Zeiss). For ParB-GFP, exposure times were 1 seconds with 32% of mercury light and for Hbs-GFP exposure times were 400 ms and using an additional 1.6x magnification. Microscopy images were deconvolved using softWoRx 3.6.0 (Applied Precision). For time course experiments cells were taken from liquid cultures at the indicated time points, spotted onto agar pads and directly imaged under the microscope. For immunoblotting samples were taken at given time intervals and protein extracts prepared using glass bead disruption and TCA extraction. Blots were probed using antibodies against the Pk tag (α-V5; Serotec) or rabbit serum directed against *B. subtilis* MreB protein.

Imaging of vegetative cells

Cells were grown overnight in SMG medium at 37 °C and diluted 100-fold into fresh SMG. After 3 hr of growth 0.5 µl of the culture at OD_{600} ~ 0.02 were spotted onto LB agar pads (in 1 % agarose) with or without HU pre-warmed at 37 °C. Slides were transferred to the environmental chamber at the microscope and incubated at 30 °C. Images were taken on a Zeiss Axio Observer Z1 microscope equipped with a Hamamatsu OrcaR^2 camera. A Plan-

Apochromat 100x/1.4 Oil Ph3 objective (Zeiss) was used. To monitor GFP fluorescence the filterset 38 HE eGFP shift free (Zeiss) was used. Digital images were acquired with Zen software (Zeiss). For the first 40 min cells were imaged every 10 min using phase contrast only to monitor growth. Afterwards, GFP and PC images were taken every 10 min for at least 180 min.

Growth assays in microtitre plates

Cells were grown exponentially in SMG medium at 37 °C. Cultures were diluted 9-fold into 200 µl pre-warmed LB medium supplemented with different concentration of antibiotics in microtitre plates (Costar #3596). Plates were incubated at 37 °C with continuous shaking in a Thermo Multiskan FC plate reader. Growth was determined by light scattering at 620 nm and average growth curves were calculated from at least three replicate samples per tested condition.

Determination of *oriC*/*ter* ratio

Cells were grown to mid-exponential phase in SMG medium at 37 °C, diluted eleven-fold into fresh SMG or LB medium supplemented with inhibitors and grown for five generations. To normalize *oriC*/*ter* ratios an exponential LB culture was treated for 4 hr in 200 µg/ml chloramphenicol for replication run-out. 20 ml LB culture (OD_{600} ~ 0.1) and 100 ml SMG culture (OD_{600} ~ 0.02) were poured onto ice and harvested by centrifugation and filtration, respectively. Genomic DNA was prepared using the Wizard genomic DNA kit (Promega). gDNA samples were diluted to 1 ng/µl and used as templates for qPCR as described [S6]. Mean and standard deviation were calculated from at least three independent samples.

Replication profile determination

Cells were grown in mid-exponential phase in LB medium at 37°C. HU, HB-EMAU or DMSO was added to aliquots of the culture and incubated for 60 min at 37°C. Cells were poured onto ice and harvested by centrifugation. gDNA was prepared using Promega Wizard gDNA kit. A shotgun library was prepared by the Max Planck Genome Centre Cologne and one million molecules were sequenced on an Illumina HiSeq2500. Reads were mapped to the reference genome using web-based platform Galaxy and analyzed using SeqMonk.

Supplemental References

S1. Anderson, L.M., and Bott, K.F. (1985). DNA packaging by the Bacillus subtilis defective bacteriophage PBSX. Journal of virology *54*, 773-780.

S2. Wood, H.E., Dawson, M.T., Devine, K.M., and McConnell, D.J. (1990). Characterization of PBSX, a defective prophage of Bacillus subtilis. Journal of bacteriology *172*, 2667-2674.

S3. Burmann, F., Shin, H.C., Basquin, J., Soh, Y.M., Gimenez-Oya, V., Kim, Y.G., Oh, B.H., and Gruber, S. (2013). An asymmetric SMC-kleisin bridge in prokaryotic condensin. Nature structural & molecular biology *20*, 371-379.

S4. Meile, J.C., Wu, L.J., Ehrlich, S.D., Errington, J., and Noirot, P. (2006). Systematic localisation of proteins fused to the green fluorescent protein in Bacillus subtilis: identification of new proteins at the DNA replication factory. Proteomics *6*, 2135-2146.

S5. Gruber, S., and Errington, J. (2009). Recruitment of condensin to replication origin regions by ParB/SpoOJ promotes chromosome segregation in B. subtilis. Cell *137*, 685-696.

S6. Murray, H., and Errington, J. (2008). Dynamic control of the DNA replication initiation protein DnaA by Soj/ParA. Cell *135*, 74-84.

S7. Kawai, Y., Moriya, S., and Ogasawara, N. (2003). Identification of a protein, YneA, responsible for cell division suppression during the SOS response in Bacillus subtilis. Molecular microbiology *47*, 1113-1122.
